# Negative Glucocorticoid Response-Like Element from the First Intron of the *Chicken Growth Hormone* Gene Represses Gene Expression in the Rat Pituitary Tumor Cell Line

**DOI:** 10.3390/ijms17111863

**Published:** 2016-11-09

**Authors:** Jing-E. Ma, Qian-Qian Lang, Feng-Fang Qiu, Li Zhang, Xiang-Guang Li, Wen Luo, Juan Wang, Xing Wang, Xi-Ran Lin, Wen-Sheng Liu, Qing-Hua Nie, Xi-Quan Zhang

**Affiliations:** 1 Department of Animal Genetics, Breeding and Reproduction, College of Animal Science, South China Agricultural University, Guangzhou 510642, China; magiel1018@stu.scau.edu.cn (J.-E.M.); langqianqian@stu.scau.edu.cn (Q.-Q.L.); qff_1804@163.com (F.-F.Q.); zhangli761101@163.com (L.Z.); 13751897799@163.com (X.-G.L.); luowen729@163.com (W.L.); wangjuan901226@126.com (J.W.); wangxing635@163.com (X.W.); 15626195391@163.com (X.-R.L.); wsliu@scau.edu.cn (W.-S.L.); nqinghua@scau.edu.cn (Q.-H.N.); 2 Guangdong Provincial Key Lab of Agro-Animal Genomics and Molecular Breeding, South China Agricultural University, Guangzhou 510642, China; 3 Key Lab of Chicken Genetics, Breeding and Reproduction, Ministry of Agriculture, South China Agricultural University, Guangzhou 510642, China

**Keywords:** intron 1, gene expression, *chicken growth hormone* gene

## Abstract

The effects of introns, especially the first intron, on the regulation of gene expression remains unclear. Therefore, the objective of the present study was to investigate the transcriptional regulatory function of intron 1 on the *chicken growth hormone* (*cGH*) gene in the rat pituitary tumor cell line (GH4-C1). Transient transfection using first-intron-inserted *cGH* complete coding sequences (CDSs) and non-intron-inserted *cGH* CDS plasmids, quantitative RT-PCR (qRT-PCR) and western blot assays were used to detect the expression of *cGH*. The reporter gene assay was also used to investigate the effect of a series of fragments in the first intron of *cGH* on gene expression in GH4-C1. All of the results revealed that a 200-bp fragment located in the +485/+684 region of intron 1 was essential for repressing the expression of *cGH*. Further informatics analysis showed that there was a cluster of 13 transcriptional factor binding sites (TFBSs) in the +485/+684 region of the *cGH* intron 1. Disruption of a glucocorticoid response-like element (the 19-nucleotide sequence 5′-AGGCTTGACAGTGACCTCC-3′) containing a T-box motif (TGACCT) located within this DNA fragment increased the expression of the reporter gene in GH4-C1. In addition, an electrophoretic mobility shift assay (EMSA) revealed a glucocorticoid receptor (GR) protein of rat binding to the glucocorticoid response-like element. Together, these results indicate that there is a negative glucocorticoid response-like element (nGRE) located in the +591/+609 region within the first intron of *cGH*, which is essential for the down-regulation of *cGH* expression.

## 1. Introduction

Introns, particularly the first intron, have large positive or negative effects on the regulation of gene expression in mammalian and fish cells [1,2]. In physiologically relevant cell lines, reporter gene assays on 107 genes in humans revealed that most regulatory elements were in the proximal regulatory promoter regions of the genes, which were located in the 5′-flanking and the intron 1 sequences [3]. Some of these first introns contained conserved sequences, which were the binding motifs of necessary functional transcription factors. When these regions were deleted by homologous recombination in knock-out mice, the mRNA level of a gene was diminished [4]. Most intron 1 sequences were positively correlated with expression of the reporter gene, and the first intron of a gene could stimulate the transcriptional activity of the gene [5,6,7,8,9,10,11]. Conversely, a small fraction of the intron 1 sequences was associated with a reduction in reporter gene transcriptional levels [12,13]. Transfection assays on the transcriptional regulation of the *gilthead sea bream* (*Sparus aurata*) *growth hormone* (*saGH*) gene implied that long growth hormone (GH) introns may influence *GH* expression in vivo [12]. Studies on the transcriptional regulation of *GH* genes have been reported mainly in mammals, birds and fish [12,14,15,16,17,18,19,20]. Previous studies have demonstrated that transcriptional factor binding sites (TFBSs) in the *GH* promoter and introns may regulate its transcription by binding with the corresponding transcription factors [15,20,21]. There might also be regulators in the introns that were recognized by the pituitary-specific transcription factor GHF-1/Pit-1 in regulating *GH* transcription [20,21,22,23]. The regulatory elements in the first intron might also cooperate to regulate the cell-type specific expression of the *GH* at the transcriptional level [14,23,24]. *GH* mRNA could be regulated by corticosterone (Cort) in vitro through a degenerate glucocorticoid response element (dGRE) half site in pituitary cells from embryonic rats or chicken. In rat, the function of dGRE was found to enhance the *rat growth hormone* (*rGH*) gene activity in the 5′-flanking sequences of *rGH*, similar to the role of glucocorticoid receptor *cis*-elements in the flanking region that could induce *chicken growth hormone* (*cGH*) gene expression in chicken embryonic pituitary cells [14,17,25].

*Chicken growth hormone* gene plays a major role in regulating somatic growth in chickens, and *cGH* sequences have been characterized in previous studies [25]. The full length of the *cGH* intron 1 sequences was shown to be 915 bp (ENSGALT00000000328). Analysis of *GH* intron 1 in 26 species showed that the length of intron 1 sequences of *GH* under evolutionary selection pressure highlights a rule among mammals, birds, reptiles and fish. The length of the first intron is much longer in chicken than in mammals but is shorter than in turtles and some fish [26], which could affect the efficiency of gene transcription. The transcription efficiency is found to be lower with longer introns [27]. In other words, the difference of the length of the intron might result in the varied number of transcriptional factor binding sites (TFBSs) in these species. Further, the function of the intron 1 is diversified on the regulation of *GH* expression. The *cis*-elements in the flanking region of *cGH* were shown to be necessary for the glucocorticoid induction of *cGH* expression in chicken embryonic pituitary cells [17]. However, the function of the first intron remains rudimentary in *cGH*. It is also not clear that the role of the dGRE in regulating *cGH* expression within the first intron. This observation led us to focus on the molecular mechanisms responsible for the *cGH* expressional regulation of intron 1 in the present study. Since rat pituitary tumor cell line (GH4-C1) is similar to chicken pituitary cells, which could also produce glucocorticoid receptor (GR) protein, it is used as an alternative material to carry out the experimental assays. Several levels of regulation, including transcriptional and translational, have been considered in the cells. We have examined whether the length of the fragments and some special motifs in the first intron of *cGH* contribute to the mRNA and protein expression level of *cGH* in vitro. To this end, we cloned the intron 1 sequences of *cGH* to the pcDNA3.1 and pGL3-control vectors to demonstrate that intron 1 sequences had a pronounced influence on the reduction of *cGH* expression in vitro.

## 2. Results

### 2.1. Effect of First Intron on Chicken Growth Hormone (cGH) Complete Coding Sequences (CDSs) Expression In Vitro

**Figure 1:**
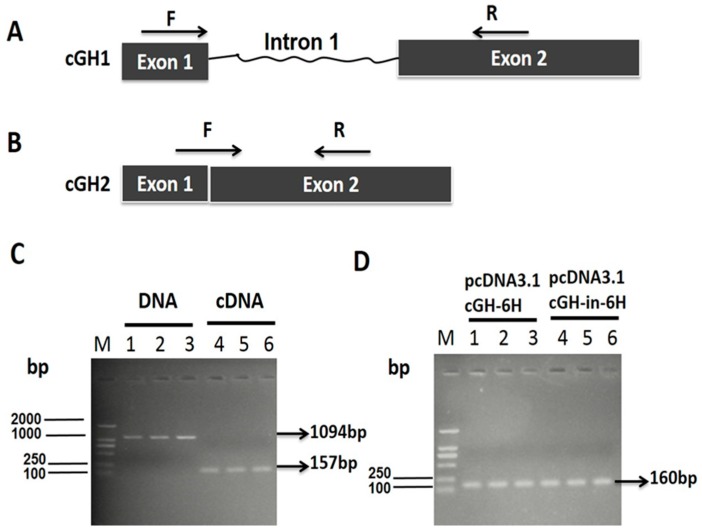
PCR product confirms the correct splicing of *chicken growth hormone* (*cGH*) intron 1 in GH4-C1 cell lines at 48 h post-transfection with pcDNA3.1cGH-6H or pcDNA3.1cGH-in-6H. (**A**) A schematic diagram showing the position of cGH1 primers in *cGH*, which are depicted by arrows. The forward primer (F) was located on exon 1, and the reverse primer (R) was located on exon 2; (**B**) a schematic diagram showing the position of cGH2 primers in the *cGH*, which are depicted by arrows. The forward primer (F) was located at the junction of exon 1 and exon 2, and the reverse primer (R) was located on exon 2; (**C**) an electrophoresis map showing the PCR product generated from the GH4-C1 cell lines post-transfection with pcDNA3.1cGH-in-6H. cGH1-F and cGH1-R were used as the primers, which are depicted in (**A**). DNA or cDNA from the GH4-C1 was used as a template, marked by the white bold characters; (**D**) an electrophoresis map showing PCR products generated from the GH4-C1 post-transfection with pcDNA3.1-cGH-6H or pcDNA3.1-cGH-in-6H. cGH2-F and cGH2-R were used as the primers, as depicted in (**B**). The temples were cell cDNA from the GH4-C1 transfected with pcDNA3.1-cGH-6H or pcDNA3.1-cGH-in-6H, which are marked by black bold characters. The lanes show the cell samples chosen at random from the GH4-C1 in (**C**,**D**).

We isolated a 915-bp intron 1 sequence (+55/+969) from the chicken genomic library by using chicken DNA as a template. The 651-bp complete coding sequences (CDSs) was directly amplified by the PCR technique using cDNA as a template. The fragment containing the CDSs and the first intron was amplified by overlapping PCR using both genomic DNA and cDNA as templates. The plasmid pcDNA3.1cGH-6H contained the 651-bp CDSs, whereas the 915-bp intron 1 sequence was inserted into the pcDNA3.1cGH-in-6H between the exon 1 and exon 2. These vectors, which were constructed through the linkage between the PCR fragment and the plasmids, were tested by the restriction enzyme reaction. The electrophoresis maps showed that the vectors were correct (Figure S1A). To observe the efficient splicing of the *cGH* intron 1 in GH4-C1 cell lines at 48 h post-transfection with pcDNA3.1cGH-in-6H, it was detected by PCR amplification whether the intron 1 sequence was retained in the transcriptional products. DNA and RNA were prepared from the transfected cells. Exogenous sequences were identified using DNA or cDNA as the template and by designed cGH1 primers, as shown in Figure 1A. The PCR amplification results presented in Figure 1C show that the length of the product was a 1094-bp fragment based on DNA as a template but when cDNA was used as a template, the length of the product was 157 bp. This showed that the transcriptional products did not retain the intron 1 sequence in the GH4-C1 transfected with pcDNA3.1cGH-in-6H. To further analyze the accurate splicing of intron 1 in the *cGH*, we investigated part of its mRNA sequence in the GH4-C1 transfected with pcDNA3.1cGH-6H or pcDNA3.1cGH-in-6H plasmids (Figure 1D). Using cell cDNA as the template, the cGH2 primers shown in Figure 1B were designed to detect whether the first intron spliced correctly. We cloned a 160-bp fragment of the *cGH* in the cell group transfected with pcDNA3.1-cGH-6H or pcDNA3.1-cGH-in-6H plasmids (Figure 1D). These results showed that the *cGH* intron 1 had been spliced correctly and efficiently in the GH4-C1.

**Figure 2:**
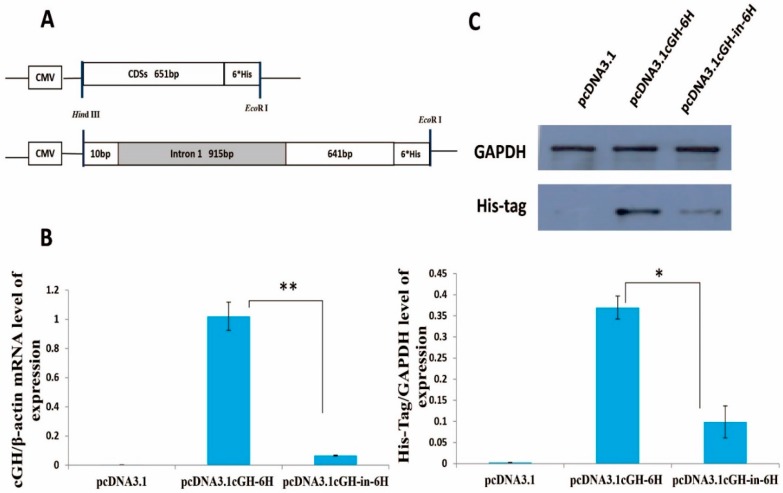
*cGH* mRNA and protein expression following transfection of the GH4-C1 cell lines. (**A**) Diagrammatic representation of the pcDNA3.1-cGH-6H and pcDNA3.1-cGH-in-6H vectors; (**B**) the mRNA expression level with the qRT-PCR test after the GH4-C1 was treated with vectors for 48 h. The expression level of chicken GH mRNA transfection with pcDNA3.1cGH-in-6H was only 7% relative to pcDNA3.1cGH-6H; (**C**) the protein expression level with the western blot test after the GH4-C1 treated with vectors for 48 h. The expression level of cGH protein transfection with pcDNA3.1cGH-in-6H was only 27% relative to pcDNA3.1cGH-6H. Each data point represents mean ± SEM of three replicates. Asterisks indicate significant difference when comparing the two indicated constructs, * means *p* < 0.05, ** means *p* < 0.01. CMV, cytomegalovirus; CDSs, complete coding sequences.

To analyze the possible effect of the intron 1 sequence on *cGH* expression in vitro, GH4-C1 cells were transduced with the plasmids containing cGH-6H or cGH-in-6H sequences linked to a cytomegalovirus (CMV) promoter, which was chosen to ensure the expression of the CDSs [28,29,30]. A diagrammatic representation of the pcDNA3.1-cGH-6H and pcDNA3.1-cGH-in-6H vector is shown in Figure 2A. The results shown in Figure 2B represent two constructs with three replicas for each one and were normalized to *cGH* mRNA activity of pcDNA3.1cGH-6H. The two vectors directed the expression of the *cGH* CDSs in the transfected cells; the cGH-in-6H plasmid decreased the expression of the *cGH* CDSs in the GH4-C1 cell line compared to the cGH-6H construct. The expression level of *cGH* mRNA transfection with pcDNA3.1cGH-in-6H was only seven percent relative to pcDNA3.1cGH-6H (*p* < 0.01). This result suggested that the first intron of the *cGH* contributed to the reduced level of *cGH* transcripts in vitro.

To account for the expression level of cGH protein differences resulting from the two constructs, the ends of the DNA fragments named cGH-6H and cGH-in-6H were filled in by 6× His-tagged protein sequences, which could be detected easily. The mean expression level of the 6× His was recorded based on western blot assays. The expression level of chicken GH protein transfection with pcDNA3.1cGH-in-6H was only 27% relative to pcDNA3.1cGH-6H (Figure 2C), indicating that the region between +55 and +969 of the *cGH* had a sequence that might down-regulate *cGH* expression in pituitary cells.

### 2.2. Effect of Intron 1 Fragments on Reporter Gene Expression In Vitro

Further, we cloned a line of seven intron 1 fragments from the *chicken GH* gene intron sequence; the lengths were 915, 598, 485, 286, 200, 715 and 897 bp. The sequences were inserted in the pGL3-cGH-ins. All of the fragments were tested by sequencing technology. The vectors, which were constructed through the linkage between the PCR fragment and the plasmids, were tested by the restriction enzyme reaction. The electrophoresis maps showed that the vectors were correct (Figure S1B–D).

**Figure 3:**
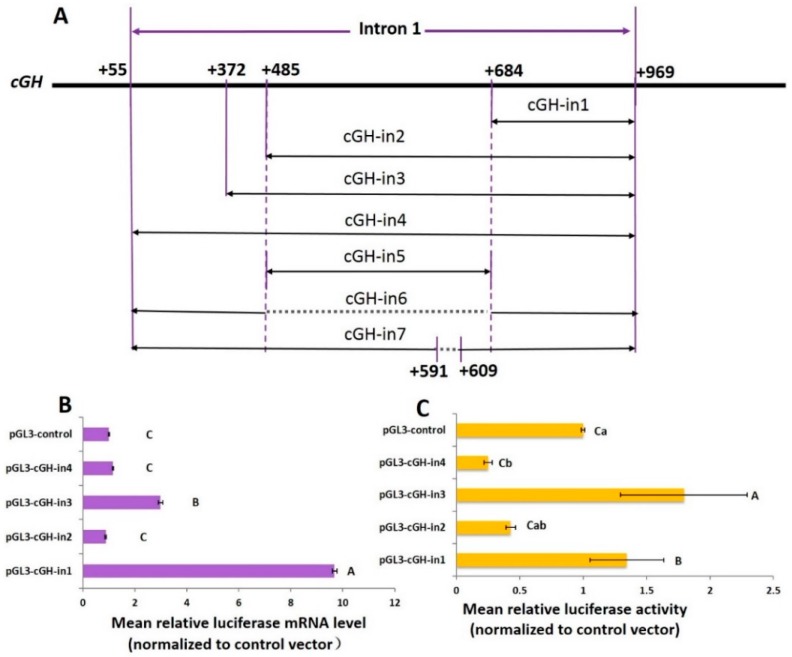

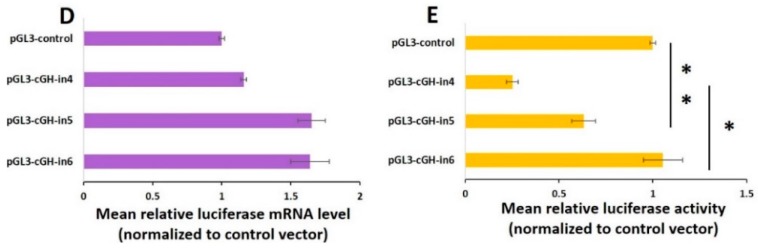
The effect of first intron fragments on promoter activity in GH4-C1. Reporter gene mRNA/protein expression level was measured 48 h after transient transfections in GH4-C1 cells. The mean relative luciferase mRNA/protein level was defined as the ratio between the firefly luciferase gene and the Renilla luciferase gene. (**A**) Diagrammatic representation of the fragments which inserted into the pGL3-control. The six fragments were cloned in the left orientation relative to the SV40 promoter. The solid lines represent the corresponding location of each fragment, while the dotted lines represent the sequences that were knocked out in the relevant fragment. The DNA region is located upstream of the transcription start site (designated +1); (**B**) the mean relative luciferase mRNA transfected with the four plasmids (pGL3-cGH-in1, pGL3-cGH-in2, pGL3-cGH-in3 and pGL3-cGH-in4); (**C**) the mean relative luciferase activity transfected with the four plasmids (pGL3-cGH-in1, pGL3-cGH-in2, pGL3-cGH-in3 and pGL3-cGH-in4); (**D**) the mean relative luciferase mRNA transfected with the three plasmids (pGL3-cGH-in4, pGL3-cGH-in5 and pGL3-cGH-in6); (**E**) the mean relative luciferase activity transfected with the three plasmids (pGL3-cGH-in4, pGL3-cGH-in5 and pGL3-cGH-in6). The purple or yellow columns represent the values, and bars indicate the standard error. Different lowercase or uppercase letters indicate a significant difference (*p* < 0.05 or *p* < 0.01) when comparing the two indicated groups. Asterisks indicate a significant difference when comparing the two indicated groups, * means *p* < 0.05, ** means *p* < 0.01.

To investigate the possible effect of different regions in the intron 1 sequence on promoter activity, four constructs, i.e., pGL3-cGH-in1 (+684/+969 construct), pGL3-cGH-in2 (+485/+969 construct), pGL3-cGH-in3 (+372/+969 construct), pGL3-cGH-in4 (+55/+969 construct), were tested (Figure 3A). These four constructs were transfected into the GH4-C1. The results are shown in Figure 3B,C. Each one was normalized to control vector. In the transfected cells, the four vectors directed the expression of the reporter gene. A higher response was obtained with the +684/+969 construct and the +372/+969 construct. The firefly luciferase mRNA levels of the +684/+969 construct and +372/+969 construct vectors increased 9.7- and 3.0-fold compared to pGL3-Control, respectively. Conversely, firefly luciferase mRNA was blocked by transfection with the +485/+969 construct compared to pGL3-Control. The luciferase mRNA level of the +55/+969 construct was almost the same as that of pGL3-Control (Figure 3B).

As shown in Figure 3C, the +684/+969 construct and +372/+969 construct exhibited increased luciferase activity by 1.3- and 1.8-fold, respectively, relative to the pGL3-Control. Compared to pGL3-Control, the +485/+969 construct and +55/+969 construct exhibited decreased luciferase activity by 43% and 25%, respectively. These results suggested that there might be negative regulatory element binding sites in the +485/+684 region of the *cGH* intron 1, whereas there might be elements that increase *cGH* expression in the +372/+485 and +684/+969 regions of the *cGH* intron 1. The +55/+969 Luc plasmid resulted in a reduction of translational luciferase activity. This finding suggested that the ongoing luciferase protein synthesis was down-regulated by the +55/+372 region in *cGH*. Because the activities of the +485/+969 Luc plasmid and +55/+969 Luc plasmid were not significantly different, we argue that the +485/+969 region of the *cGH* intron 1 is mainly associated with repressed regulation, which contains an important down-regulated region of +485/+684.

To delineate the DNA elements in the +485/+684 region that are responsible for *cGH* expression in vitro, we isolated the +485/+684 region from the *cGH* up to the region and injected the sequence into the pGL3-Control construct, which was called pGL3-cGH-in5 (Figure 3A). The data revealed that the pattern of +485/+684 construct expression in transduced cells was similar to that seen after transfection of the +55/+969 construct containing the entire intron 1 sequence, suggesting that the +485/+684 region was the key fragment for regulating the reporter gene expression in GH4-C1 (Figure 3D,E). We deleted this intronic region in the first intron for further study and pGL3-cGH-in6 was constructed (Figure 3A). As shown in Figure 3D,E, after removing the +485/+684 region, the expression of the reporter gene was markedly changed compared with +55/+969 construct.

### 2.3. Transcriptional Factor Binding Sites (TFBSs) in Intron 1 of the cGH Gene

The full length of the *cGH* intron 1 was 915 bp, in which several TFBSs were identified. There were 32 types of TFBSs within the first intron fragment, including the enhancers, i.e., CCAAT/enhancer binding protein beta (CEBPB), CCAAT/enhancer binding protein alpha (CEBP), cAMP activated protein (CAP), GATA binding elements (GATA1), LIM-only protein 2 (LMO2COM), myeloid zinc finger 1 (MZF1), simian-virus-40-protein-1 (SP1), basic helix–loop–helix transcription factors (USF), the repressor, acute myeloid leukemia-1 (AML1), and the enhancers or repressor GRE [17,31]. There were also some elements with unclear functions, i.e., AP1, replication initiator 1 (AP4), AP1FJ, c-Ets-1 (CETS1P54), c-Rel (CREL), caudal type homeobox 1 (CDXA), DELTAEF1, cyclin E/E2F (E2F), E47, GATA3, GATA2, hepatocyte nuclear factor 3β (HNF3β), ikappa B kinase-like 2 (IK2), c-Myb (MYB), MYOD, cardiac-specific homeobox protein (NKX25), OCT1, P53, sex-determining region Y (SRY), STAT, TATA, and TST1 [32,33,34,35,36] (Table S1). The 5′-distal region of +55/+372 in the *cGH* contained a cluster of twenty TFBSs and highly conserved near-consensus TFBSs (score = 1.000) for the transcriptional factor, which was acute myeloid leukemia-1 (AML1). There was a cluster of eight TFBSs in the +372/+485 region and thirteen TFBSs in the +684/+969 region (Table 1). Nevertheless, the cluster of 13 TFBSs was found between +485 and +684 in the *cGH* intron 1 (Figure S2). The degenerate GRE was found in this region, which had been proven to induce *GH* expression in cultures of pituitary cells in mouse, human and chicken *GH* genes [14,15,17]. However, the sequence TGACCT was identical to the second motif of the GRE between +591 and +609, which was also part of the repetitive units of the polymorphic minisatellite saGHFIM [12], thus suggesting that the putative GRE in the +485/+684 region might be involved in the repression.

### 2.4. Role of the Intronic Negative Glucocorticoid Response Element (nGRE) in Reporter Gene Expression In Vitro

**Figure 4:**
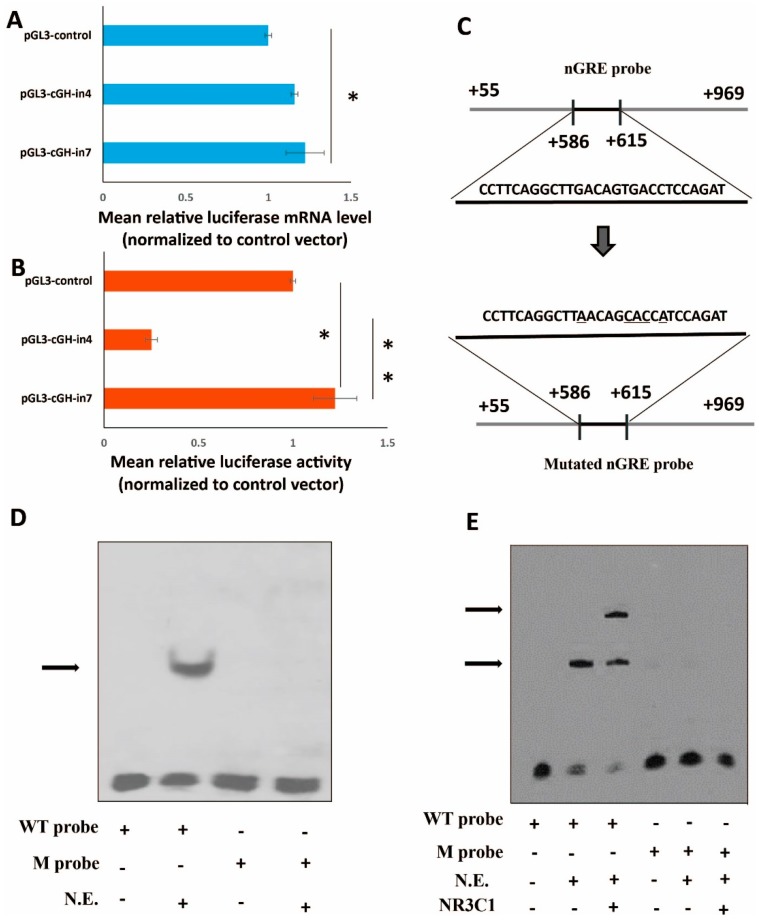
Role of the intronic negative glucocorticoid response element (nGRE) in reporter gene expression in GH4-C1. (**A**) The mean relative luciferase mRNA transfected with the plasmid pGL3-cGH-in4 and pGL3-cGH-in7; (**B**) the mean relative luciferase activity transfected with the plasmid pGL3-cGH-in4 and pGL3-cGH-in7. Asterisks indicate a significant difference when comparing the two indicated groups, * means *p* < 0.05, ** means *p* < 0.01; (**C**) A diagrammatic representation of the nGRE probe and mutated probe, the underlined sequences showed the mutant sequences in the mutated probe; (**D**) A representative image of nuclear protein extracts from GH4-C1 cells incubated with the wild-type (WT) nGRE probe or a mutated (M) nGRE probe (*n* = 3 separate experiments), the arrow showed the lower-shifted band; (**E**) GR from GH4C1 cells was tested for binding to the nGRE probe. Electrophoretic mobility shift assay (EMSA) gel was transferred to a polyvinylidene difluoride membrane, which was detected by NR3C1 antibody. The arrows showed the lower-shifted bands. Blots represent results from three replicate experiments.

To characterize the function of the DNA regulatory element in the region, the +591/+609 region in intron 1 was deleted and the mutant fragment increased the reporter gene expression in GH4-C1. Compared with pGL3-control, both the firefly luciferase mRNA level of pGL3-cGH-in7 (Figure 4A) and luciferase activity of the construct (Figure 4B) significantly increased (*p* < 0.05), while compared to +55/+969 construct, the luciferase activity of the construct (Figure 4B) increased extremely significantly (*p* < 0.01), indicating that this intronic region was essential for the repressed regulation of *cGH*.

Because the expression studies indicated that important regulatory elements were contained within +591/+609 of the first intron, the specific DNA consensus element in this region was the motif of glucocorticoid response-like element containing TGACCT sequences, which was a potential negative glucocorticoid response element (nGRE). To determine whether the +591/+609 DNA regulatory sites were able to bind with protein factors, electrophoretic mobility shift assay (EMSA) was performed with the purified GH4-C1 cell nucleus protein. In the experiments labeled oligonucleotides were used, in which containing the nGRE located in the +586/+615 region within the first intron (Figure 4C). As shown in Figure 4D, a single DNA-protein complex was observed. Conversely, mutation of the nGRE consensus sequence functionally abolished its protein-binding affinity (Figure 4D), thus indicating the inability of mutant oligonucleotides to effectively form the protein complex. Furthermore, the proteins from the acrylamide gel were then transferred to PVDF membranes and probed for GR, which was detected in the proteins that bound the nGRE probe (Figure 4E, left). However, the GR antibody did not detect any proteins that bound the mutated probe (Figure 4E, right).

### 2.5. Effect of the Length of GH Intron 1 Fragments on Reporter Gene Expression In Vitro

Generally, the number of the TFBSs in an intron corresponded to the length of the intron, which had various functions. Thus, the length of an intron might contribute to regulating the transcriptional activity by binding with various TFBSs. We further assessed the effect of a short and a long first intron fragment on reporter gene expression in vitro using transient transfection into the GH4-C1 cell line. Seven constructs were tested, as illustrated schematically in Table 2. The pGL3-cGH-in4 with the longest intronic fragment (915 bp) were in forward orientation, in contrast to the SV40 promoter, whereas pGL3-cGH-in5 had the shortest intronic fragment (200 bp). Relative luciferase activity (normalized to Renilla luciferase activity) was proportional to control (pGL3-Control) expression, regarded as 100%. For pGL3-cGH-in1, pGL3-cGH-in3, pGL3-cGH-in4, pGL3-cGH-in6 and pGL3-cGH-in7, the effect of the longest intron was obvious (there was a significant reduction in average luciferase activity relative to others), whereas the shortest intron showed a significant increase in average luciferase activity relative to others. The long intron fragment had a repressing effect on gene expression compared to the control plasmid or a short intron fragment. These results suggested that the length of intron 1 that might influence gene expression.

## 3. Discussion

Although the first intron is widely accepted as a consequence of a regulatory region, its significance has been studied only occasionally. However, recent evidence indicated that the first intron of the *pig GH* gene (*pGH*) increased *pGH* expression [18]. Here, we isolated the first intron of the 915-bp DNA fragment located in the chicken growth hormone gene and investigated its function with regard to *cGH* expression in vitro. Analyses of this DNA fragment in the GH4-C1 cell lines from pituitary tissues of rat demonstrated that the expression of *cGH* was down-regulated by intron 1 on both the transcriptional and translational levels, which might result from a slower splicing reaction and regulatory element binding sites in intron 1 [37,38]. Therefore, the first intron of *cGH* might be a critical negative fragment that determines the expression and function of the *cGH* protein in vivo.

Consistent with a reduction in *cGH* protein by intron 1, the subsequent expressional experiments revealed markedly reduced reporter gene translational expression in GH4-C1, but this was not significantly regulated in transcriptional activity. This was further confirmed by the expressional profile of the +485/+684-reporter gene construct. These data indicated that the process of intron-splicing led to the repression of *cGH* transcriptional activity and that the +485/+684 region located in the first intron had a negative effect on the expression of *cGH* in vitro. On the basis of this observation, several potential *cis*-acting elements, such as SRY, CETS1P54, NKX25, E47, AML1 and GRE, were aligned among this DNA fragment, which revealed that the sequence between +591 and +609 might be involved in the repression; this was the motif of the nGRE with TGACCT sequences. In agreement with our current data, these subunits in the first intron region have been viewed as essential elements for expression of the *human GH* gene (*hGH*) or *saGH* [12,15]. Transient transfection assays showed that the polymorphic minisatellite (saGHFIM) found in the first intron of *saGH* on gene expression in four cell lines, which consists of a 13–17 bp repeat unit with the sequence TGACCTGTCTSTCTCTC, could correspond to a negative regulatory structure [12]. Previous studies also indicated that the human, rainbow trout and chicken growth hormone genes contained a structure within their introns that could function as a GRE and demonstrated that the TFBS of GRE present in intron 1 of the *GH* significantly increased the activity of the reporter gene in cells [15,17,20]. However, our deletion studies have identified the +591/+609 region to significantly decrease the expression of *cGH*. We further showed that this intronic DNA fragment succeeded in specifically binding to the protein complex and that its function clearly diminished with the mutation of the 5-bp core element. Therefore, we argue that the nGRE motif could down-regulate the expression of the *cGH* from the first intron position and could function as a translation-specific repressor in downstream locations.

Previous studies in diverse organisms, including *Homo sapiens*, *Drosophila melanogaster*, *Caenorhabditis elegans*, *Saccharomyces cerevisiae*, and *Arabidopsis thaliana*, indicated that there are negative relationships among expression level and gene size, CDS length and intron length [38]. However, it remains unclear whether the length of introns affects the gene expression. In this context, the roles of the reporter gene and short or long intron 1 fragments were determined in vitro using the pituitary cell lines. The results revealed a significant reduction in luciferase activity by the construct containing the long fragment located in the first intron. The highest repression of +55/+969 construct was noticeable in the experiments relative to the control vector. Moreover, the repression was also significant compared to that observed for the two short fragments of +485/+684 and +485/+969, in which the key negative regulatory region could play a repressing role in regulating gene expression.

This repressing effect was seen in the over-expression results and in the reporter gene data, and several conclusions can be drawn from these experiments. First, high repression generated by the first intron occurs in rat pituitary cell lines, indicating that this regulation sequence is involved in a vertebrate-conserved mechanism of gene regulation. Second, TFBSs determine the level of repression in both transcriptional and translational processes. The length of the first intron could also contribute to regulating the transcriptional and translational activity through the recruitment of certain transcriptional factors of different quantities and functions. Additional experiments highlighted the contribution of the nGRE motif in the repression of *cGH* expression.

We have known for decades that intron 1 can regulate *GH* expression in cell lines [12,14,15,17,18,20,25]. However, the mechanism underlying this response has remained elusive. In the present study, we took advantage of the ability to identify a region of *cGH* required for gene regulation (the important nGRE) and found that suppression activity was greater with an increased number of TFBSs. These findings have implications for regulation of the GH gene and growth axis during cell growth, sexual maturation and immune functions in vertebrates.

## 4. Materials and Methods

### 4.1. Construction of Plasmids

Genomic DNA and RNA templates were isolated from the blood and pituitaries of chickens, respectively. The fragment of cGH-6H only contained *cGH* complete coding sequences (CDSs), whereas cGH-in-6H consisted of *cGH* CDSs plus the first intron of *cGH*. Each fragment was amplified using the primers that contained restriction enzyme sequences (Table S2). The ends of the two DNA fragments (Table S3) were filled in by sequences encoding six repeating histidines. This His-tag was used to evaluate protein expression of *cGH*.

The plasmids named pcDNA3.1cGH-6H and pcDNA3.1cGH-in-6H were constructed by replacing sequences between the Hind ІІІ (+912) and EcoR І (+953) sites of the pcDNA3.1(+) vector (Invitrogen) with the cGH-6H and cGH-in-6H sequences generated by PCR. Construction of the pcDNA3.1cGH-6H and pcDNA3.1cGH-in-6H was based on the design of a previous study [18].

The plasmids named pGL3-cGH-in1, pGL3-cGH-in2, pGL3-cGH-in3, pGL3-cGH-in4 and pGL3-cGH-in5 were constructed by replacing sequences between the *Kpn* І (+5) and *Xho* I (+32), or *Nhe* I (+21) sites of the pGL3-Control vector (Invitrogen), with the sequences of several intron 1 fragments in *cGH*; i.e., +684/+969, +485/+969, +372/+969, +55/+969 and +485/+684, generated by PCR using primers that contained restriction enzyme sequences. Deletion constructs were made using the pGL3-cGH-in4 as the template in PCR amplification, known as pGL3-cGH-in6 and pGL3-cGH-in7. The primers and restriction enzymes are listed in Table S4.

### 4.2. Cell Cultures

GH4-C1 were grown in a 1640 medium (Gibco, Grand Island, NY, USA) supplemented with 10% FBS (Hyclone, Logan, UT, USA) and 0.2% penicillin/streptomycin (Invitrogen, Carlsbad, CA, USA).

### 4.3. Cell Transfection

The cell line was transfected with the following nine reconstructed vectors of pcDNA3.1cGH-6H, pcDNA3.1cGH-in-6H, pGL3-cGH-in1, pGL3-cGH-in2, pGL3-cGH-in3, pGL3-cGH-in4, pGL3-cGH-in5, pGL3-cGH-in6 and pGL3-cGH-in7. Briefly, one day before transfection, cells were trypsinized and seeded onto 24-well plates (Corning Costar Corporation, Cambridge, MA, USA) in the aforementioned growth medium, excluding antibiotics. After a 24-h period for attachment, the cells were transfected with uncut plasmids using Lipofectamine 3000 (Invitrogen, Carlsbad, CA, USA) as recommended by the manufacturer. The Renilla luciferase plasmid (pRL-TK) was co-transfected with pGL3 constructs pGL3-cGH-in1, pGL3-cGH-in2, pGL3-cGH-in3, pGL3-cGH-in4, pGL3-cGH-in5, pGL3-cGH-in6, pGL3-cGH-in7 or pGL3-Control, which was used for normalization. The plasmids to be transfected were diluted in sterile OPTI-MEM I Reduced Serum Medium (Gibco, Grand Island, NY, USA) to a concentration of 0.6 μg per well for pGL3 constructs and 0.012 μg per well for the Renilla luciferase plasmid. The cultures were incubated for 48 h after transfection.

### 4.4. RNA Isolation, RT-PCR and Real-Time Quantitative PCR (RT-qPCR)

Total RNA was isolated from tissues or cells with RNAiso reagent (Takara, Otsu, Japan), which was treated with DNase I (Takara). The quality and quantity of RNA were assessed by denatured gel electrophoresis and NanoDrop 2000c (Thermo, Waltham, MA, USA). cDNA synthesis for mRNA was performed using the PrimeScript RT reagent Kit (Perfect Real Time) (Takara). RT-qPCR was carried out in a Bio-rad CFX96 Real-Time Detection system (Bio-rad, Hercules, CA, USA). The KAPA SYBR FAST qPCR Kit (KAPA Biosystems, Wobrun, MA, USA) was used in this assay, and quantification was conducted as previously described [39]. The primer pairs used were designed by Sangon Company (Shanghai, China) (Table S5).

### 4.5. Dual-Luciferase Reporter Assay

Cell lysates transfected with pGL3 vector constructs were prepared as described above, except the lysis solution was 1× Passive Lysis Buffer (PLB, Promega, Madison, WI, USA). Cell lysates were brought to room temperature, of which 20 μL was used to carry out the luciferase assays by the Dual-Luciferase Reporter Assay System (Promega), according to the manufacturer’s instructions, using 50 µL Luciferase Assay Reagent II (LAR II) (Promega, Madison, WI, USA) and 50 μL Stop & Glo Reagent (Promega, Madison, WI, USA). The measurements were carried out as described above, and sequential readings for firefly luciferase and Renilla luciferase were recorded.

### 4.6. Immunoblotting

Immunoblotting was carried out by standard procedures, with the antibodies His-tag (Santa Cruz Biotechnology, Santa Cruz, CA, USA) and GAPDH (Bioworld, St. Louis Park, MN, USA).

### 4.7. Electrophoretic Mobility Shift Assay (EMSA)

Single-stranded (sense and antisense) 5′-biotin-labeled probes designed for the intron 1 fragment (+586/+615) of cGH (Table S6) were purchased from Sangon Biotech (Shanghai, China). Double-stranded probes were prepared by mixing equal amounts of the single-stranded cDNAs, heating to 95 °C for 5 min, then cooling to room temperature slowly. One microliter of annealed probe (50 nM final concentration) was mixed with 2.5 μg of nuclear extract proteins in the binding reaction. The binding reaction, which consisted of binding buffer (10 mM Tris, 50 mM NaCl, and 1 mM DTT, pH 7.5), 50 ng of sheared salmon sperm DNA, and 5 mM MgCl_2_, 2.5 mM DTT, 0.25% Tween 20, and 0.2% NP-40, was incubated for 20 min on ice in darkness. The DNA-protein complexes were separated by electrophoresis (3 h at 70 V) on non-denaturing 7% polyacrylamide-Tris-borate-EDTA gels and were scanned directly using the Tanon 5200 imaging system (Shanghai, China). To identify proteins that bound to the nGRE, proteins from the acrylamide gel used for the EMSA were recognized with the mouse monoclonal anti-rat GR antibody NR3C1 from Bioss (Beijing, China), as shown previously [40].

### 4.8. Statistical Analysis

Data processing was done using the statistical software package SAS 9.1.3 (SAS Institute Inc., Cary, NC, USA) and expressed as the mean ± SEM. Variance analysis was performed using the General Linear Model (GLM) procedure, based on at least three replicates for each treatment. The BLAST program (available at: http://blast.ncbi.nlm.nih.gov/Blast.cgi) on the NCBI website was used to study sequence homology. The SeqMan program of the DNASTAR software suite was used for sequence alignment, and TFBSs of the first intron in *cGH* were predicted by the websites (available at: http://www.softberry.com/berry.phtml?topic=nsite&group=programs&subgroup=promoter) and (available at: http://tfbind.hgc.jp/).

### 4.9. Ethics Standards

The experiments in this study were approved by the Animal Care Committee of South China Agricultural University (Guangzhou, China) (permitted on 1, September, 2013, permit number: SCAU#0015), and the chickens were humanely killed as necessary to ameliorate their suffering.

## Figures and Tables

**Table 1:** The potential transcriptional factor binding sites (TFBSs) in *chicken growth hormone* (*cGH*) intron 1 sequence.

Position	Name
+55/+372	AP1FJ, AP1, AP4, AML1, CAP, CDXA, CEBPB, CEBP, CREL, GATA3, GATA2, GATA1, GR, LMO2COM, MZF1, OCT1, P53, SRY, SP1, TST1
+372/+485	CAP, CDXA, CETS1P54, DELTAEF1, MYOD, NKX25, SRY, TATA
+485/+684	AP4, AML1, CAP, CETS1P54, CEBPB, DELTAEF1, E47, GRE, LMO2COM, MZF1, NKX25, SRY, USF
+684/+969	AP4, CAP, CEBPB, E2F, E47, HNF3β, IK2, LMO2COM, MZF1, MYB, STAT, USF

Position: Position on the *cGH* gene sequence. The transcription start site is set to +1.

**Table 2:** The effect of 1st intron fragments with different length on reporter gene expression level.

Groups	Length (bp)	Mean mRNA Expression Level ± SEM	Mean Protein Expression Level ± SEM
pGL3-cGH-in4	915	1.159 ± 0.0201	0.252 ± 0.0313
pGL3-cGH-in7	897	1.716 ± 0.1975	1.223 ± 0.3150
pGL3-cGH-in6	715	1.638 ± 0.1396	1.054 ± 0.1044
pGL3-cGH-in3	598	2.992 ± 0.0904	1.79 ± 0.4998
pGL3-cGH-in2	485	0.878 ± 0.0238	0.429 ± 0.0386
pGL3-cGH-in1	286	9.681 ± 0.0885	1.344 ± 0.2907
pGL3-cGH-in5	200	1.649 ± 0.1000	0.632 ± 0.0640
pGL3-control	0	1.007 ± 0.0224	1.003 ± 0.0436

Data processing was done using the statistical software package SAS 9.1.3 (SAS Institute Inc., Cary, NC, USA) and expressed as the mean ± SEM.

## Supplementary Materials

Supplementary materials can be found at www.mdpi.com/1422-0067/17/11/1863/s1.
