# Supplementary material for: Negative Glucocorticoid Response-Like Element from the First Intron of the Chicken Growth Hormone Gene Represses Gene Expression in the Rat Pituitary Tumor Cell Line
Source: Int J Mol Sci. 2016 Nov 9;17(11):1863. doi: 10.3390/ijms17111863 (PMC5133863; doi:10.3390/ijms17111863)
Supplement: Supplementary file 1 [file ijms-17-01863-s001.pdf]

Supplementary Materials: Negative Glucocorticoid Response-Like Element from the First Intron of the *Chicken Growth Hormone* Gene Represses Gene Expression in the Rat Pituitary Tumor Cell Line

Jing-E Ma, Qian-Qian Lang, Feng-Fang Qiu, Li Zhang, Xiang-Guang Li, Wen Luo, Juan Wang, Xing Wang, Xi-Ran Lin, Wen-Sheng Liu, Qing-Hua Nie and Xi-Quan Zhang

Table S1. Potential TFBSs identified in the first intron region of *cGH*.

Name	Position	Score	Subsequence/Consensus Sequence (5′→3′)	Note
P53	97 (+)	0.863130	**CTGCATGCTT**<br>**NGRCWTGYCY**	p53; RSp53; NSp53; p53as; ASp53
GRE	302 (−)<br>591 (+)	0.801618<br>0.808873	**GAAGAACAAGGCAAACCAG**<br>**AGGCTTGACAGTGACCTCC**<br>**NNNNNNCNNTNTGTNCTNN**	glucocorticoid receptor β; NR3C1; GR β
GATA3	63 (−)<br>110 (+)<br>120 (+)<br>336 (−)	0.930882<br>0.887904<br>0.884803<br>0.887461	**CTTTATCTC**<br>**GTGATGGGA**<br>**ATGATGGTG**<br>**TGCTATCAT**<br>**NNGATARNG**	GATA-3; NF-E1c (chick)
CEBPB	57 (−)<br>177 (+)<br>537 (+)<br>856 (+)<br>856 (−)	0.892521<br>0.885575<br>0.911095<br>0.887816<br>0.897341	**ACTTTGCTTTATCT**<br>**AAATGTGGCAACTT**<br>**AACTTTTGTAAGCG**<br>**ACATGGAGCAACAT**<br>**ACATGGAGCAACAT**<br>**RNRTKNNGMAAKNN**	AGP/EBP; ANF-2; C/EBP β; CRP2; H-APF-2; IL-6DBP; LAP; LAP1; NF-IL6
CEBP	57 (+)<br>175 (+)	0.894003<br>0.891841	**ACTTTGCTTTATC**<br>**AAAATGTGGCAACTTACA**<br>**NNTKTGGWNANNN**<br>**NGWNTKNKGYAAKNSAYA**	BPc; CBP; C/EBP; C/EBP α; EBP20; CCAAT/enhancer binding protein α
AML1	179 (+)<br>635 (−)<br>137 (+)	0.897865<br>0.897865<br>1.000000	**TGTGGC**<br>**GCCACA**<br>**TGTGGT**<br>**TGTGGT**	AML1; PEBP2αB; RUNX1; CBF-α2
GATA1	334 (−)<br>**109 (+)**<br>**330 (+)**	0.891238<br>**0.948667**<br>**0.904375**	**ATTGCTATCATAC**<br>**GGTGATGGGA**<br>**GGTGATTGCT**<br>**NNCWGATARNNNN**	EF1 (chick); EFgammaa; Eryf1 (chick); GATA-1; GF-1; NF-E1
NKX25	578 (+)<br>474 (−)	0.893863<br>0.950176	**GTTAATAA**<br>**CACTTGT**<br>**CWTAATTG/TYAAGTG**	Csx; Nkx-2.5; cardiac-specific homeobox protein
SRY	384 (−)<br>365 (+)<br>530 (−)<br>55 (−)	0.911240<br>0.942636<br>0.905426<br>0.923643	**GGTGATT**<br>**AAACTCA**<br>**TGAGATT**<br>**GTACTTT**<br>**AAACWAM**	SRY; sex-determining region Y gene product; TDF; testis-determining factor
MYOD	472 (+)	0.920111	**TGCACTTGTC**<br>**NNCANCTGNY**	MEF1; Myf-3 (human); MyoD; MyoD1; CMD1
GATA_C	60 (−)	0.953712	**TTGCTTTATCT**<br>**NGATAAGNMNN**	GATA-2; NF-E1b (chick)
CREL	80 (+)	0.902113	**TGGGTGTTCC**<br>**SGGRNWTTCC**	c-Rel; p82(hc-rel); p68; HIVEN86A (human)

Table S1. *Cont.*

Name	Position	Score	Subsequence/Consensus Sequence (5′→3′)	Note
AP1	197 (+)<br>242 (+)<br>242 (+)<br>243 (+)<br>243 (−)	0.902714<br>0.944190<br>0.972546<br>0.971720<br>0.979300	**AGTGACAAAGG**<br>**GCTGACTCAGG**<br>**GCTGACTCAGG**<br>**CTGACTCAG**<br>**CTGACTCAG**<br>**RSTGACTMANN**<br>**NNTGACTCANN**<br>**NTGASTCAG**	AP1; Fra-2
MYB	825 (−)	0.907469	**GCACGTTAGG**<br>**NNNAACKGNC**	c-Myb
SP1	277 (+)	0.909683	**GGGGCAGGAA**<br>**GRGGCRGGGW**	Sp1; simian-virus-40-protein-1
MZF1	108 (+)<br>570 (+)<br>827 (+)<br>941 (−)	0.914271<br>0.887528<br>0.907023<br>0.882279	**GGGTGATGGGATA**<br>**TGTCGAGGGTTAA**<br>**ACGTTAGGGGAAA**<br>**TAACCCCTCTCCT**<br>**KNNNKAGGGGNAA**	MZF-1; MZF1; myeloid zinc finger 1; ZN42; ZNF42
AP4	238 (−)<br>637 (+)<br>694 (+)<br>714 (+)<br>714 (−)<br>719 (−)	0.935800<br>0.882481<br>0.900435<br>0.960283<br>0.960283<br>0.914037	**ATGAGCTGAC**<br>**CACAGCGCAG**<br>**AGCAGCACGG**<br>**CTCAGCTGAG**<br>**CTCAGCTGAG**<br>**CTGAGCTGTT**<br>**NNCAGCTGNN**	Replication initiator 1
CAP	70 (+)<br>195 (+)<br>369 (+)<br>341 (+)<br>411 (+)<br>473 (+)<br>502 (+)<br>715 (+)<br>663 (+)<br>730 (+)<br>737 (+)	0.967965<br>0.903894<br>0.959093<br>0.934943<br>0.959093<br>0.906358<br>0.934450<br>0.900936<br>0.902908<br>0.928536<br>0.903894	**TCAGTTCT**<br>**TCAGTGAC**<br>**TCAGTTCC**<br>**TCATACGT**<br>**TCAGACTC**<br>**GCACTTGT**<br>**GCATTCCT**<br>**TCAGCTGA**<br>**TCACCCCA**<br>**CCAGTCCT**<br>**TCACCCAC**<br>**NCANNNNN**	cAMP activated protein
CDXA	63 (+)<br>381 (+)	0.929377<br>0.918097	**CTTTATC**<br>**CTTTAAA**<br>**MTTTATR**	caudal type homeobox 1
GATA2	119 (+)<br>336 (−)	0.904375<br>0.908886	**TATGATGGTG**<br>**TGCTATCATA**<br>**NNNGATRNNN**	GATA2
AP1FJ	242 (+)	0.924606	**GCTGACTCAGG**<br>**RSTGACTNMNW**	AP1
OCT1	322 (+)	0.905966	**GTTGTAATGGTGA**<br>**NNNRTAATNANNN**	OCT1
TST1	356 (+)	0.917381	**GGGATATTAAAACTC**<br>**NNKGAWTWANANTNN**	TST1
TATA	380 (+)	0.924743	**GCTTTAAAAC**<br>**NCTATAAAAR**	TATA
CETS1P54	393(+)<br>564 (+)	0.908050<br>0.903042	**GATCAGGATGATG**<br>**CTGGATGTCG**<br>**NNAMMGGAWRWNN**<br>**NCMGGAWGYN**	c-Ets-1; c-Ets-1 54; Ets1; p54; p54c-Ets-1
E47	614 (+)<br>892 (+)	0.930293<br>0.918602	**CCTACAGGTGTGTCC**<br>**ACTCACAGGTGGACAC**<br>**NSNGCAGGTGKNCNN**<br>**NNNMRCAGGTGTTMNN**	E47

Table S1. *Cont.*

Name	Position	Score	Subsequence/Consensus Sequence (5′→3′)	Note
LMO2COM	63 (−)<br>615 (+)<br>894 (+)<br>917 (+)	0.886792<br>0.901884<br>0.906864<br>0.940242	**CTTTATCTC**<br>**CTACAGGTGTGT**<br>**TCACAGGTGGAC**<br>**CCCCAGGTGCCA**<br>**NMGATANSG**<br>**SNNCAGGTGNNN**	LIM-only protein 2; RBTN2; Rhombotin-2; TTG-2
DELTAEF1	451 (+)<br>616 (−)<br>797 (−)	0.901884<br>0.918454<br>0.922027	**GATCACCTAAT**<br>**TACAGGTGTGT**<br>**GGAAGGTGAAA**<br>**NNNCACCTNAN**	areb6, bzp, deltaef1, fecd6, nil-2a, nil2a, tcf8, zeb, zfhep, zfhx1
HNF3β	703 (−)<br>797 (−)	0.909003<br>0.943460	**GTGCAAATAGGC**<br>**CCCTGTTGGCTC**<br>**NNNTRTTTRYTY**	hepatocyte nuclear factor 3 β
IK2	724 (−)	0.906741	**CTGTTCCCAGTC**<br>**NNNYGGGAWNNN**	IkappaB kinase-like 2
STAT	830 (−)	0.959163	**TTAGGGGAA**<br>**TTCCCRKAA**	STAT
USF	556 (−)<br>854 (+)<br>866 (−)<br>896 (−)	0.924187<br>0.910768<br>0.940288<br>0.919155	**TCATGTGA**<br>**CCACATGG**<br>**ACATGTGG**<br>**ACAGGTGG**<br>**NCACGTGN**	Basic helix–loop–helix transcription factors
E2F	768 (−)	1.000000	**GCGCCAAA**<br>**TTTSGCGC**	cyclin E/E2F

Position on the cGH gene sequence. The transcription start site is set to +1. + and − means forward and reverse strands that the transcription factor binds, respectively; Score: Similarity (0.0–1.0) between a registered sequence for the transcription factor binding sites and the intron 1 sequence; Subsequence: sequence from the cGH gene sequence at the position corresponding to the consensus sequence. S = C or G, W = A or T, R = A or G, Y = C or T, K = G or T, M = A or C, N = any base pair.

Table S2. Primer pairs information of chicken GH gene.

ID	Prime Name	Sequence（5′→3′）	Restriction Site
C1	cGH-DNA-F	<u>CAAGCTTG</u>ATGGCTCCAGGTACTTTGCT	HindIII
C2	cGH-cDNA-F	<u>CAAGCTTG</u>ATGGCTCCAGGCTCGTGGTT	HindIII
C3	cGH-R-6H	<u>GGAATTCC</u>TCA*atggtgatggtgatggtg*GATGGTGCAGTTGCTCT	EcoRI
C4	cGH-exon2-F1	CTCCTCTCCTCATCGCTGTG	
C5	cGH-exon2-R1	GCGGCAGTCCCAGCGTGA	
C6	cGH1-F	GAATTCAAGCTTATGGCTCCAG	
C7	cGH1-R	TCTTTATATGTCTCGGCAGC	
C8	cGH2-F	ATGGCTCCAGGCTCGTGGTT	
C9	cGH2-R	ATGTCTCGGCAGCCAGGAGG	

Italic bases in low case were His-tag sequence, sequence with underline was protective bases and enzyme loci.

Table S3. Sequence information of chicken GH gene.

Sequence Name	Temple	Primes	Length
cGH-6H	cDNA	C2 C3	685 bp
cGH-E1-IN1-E2	DNA	C1 C5	986 bp
cGH-E2-E5	cDNA	C3 C4	636 bp
cGH-in-6H	cGH-E1-IN1-E2, cGH-E2-E5	C1 C3	1600 bp

Table S4. Primer pairs information for pGL3-cGH vectors of chicken GH.

Primer Name	Title	Primer Sequence (5′→3′)	Product Size (bp)
C 1	F	ATAGGTACCGTGCCATGCCAG	286
	R	GATCTCGAGCTACAGCAGA	
C 2	F	ATAGGTACCGTCCTGCAGGCT	485
	R	GATCTCGAGCTACAGCAGA	
C 3	F	ATAGGTACCGTTCCAAGGCTTT	598
	R	GATCTCGAGCTACAGCAGA	
C 4	F	ATAGGTACCGTACTTTGCTTTATC	915
	R	GATCTCGAGCTACAGCAGA	
C 5	F	TTTCTCTATCGATAGTCCTGCAGGCTCCAGGGCATTC	200
	R	GATCTCGAGCCCGGCTGTCTGCCTTCACTGGGGTGAGAAGTGGCTGCATTACCTG	
Mut1	F	CTTGCACTTGTCCAATGCCATGCCAGCAGC	
	R	GCTGCTGGCATGGCATTGGACAAGTGCAAG	
Mut2	F	GGGTTAATAACCTTCAGATCCTACAGGTGTG	
	R	ACACCTGTAGGATCTGAAGGTTATTAACCCTC	
RVP3	F	CTAGCAAAATAGGCTGTCCC	
GLP2	R	CTTTATGTTTTTGGCGTCTTCCA	

Sequence with underline was protective bases and enzyme loci.

Table S5. Primers for qRT-PCR.

mRNA Target	Title	Primer Sequence (5′→3′)	AccessionN. in GenBank
cGH	F	TGCCGAGACATATAAAGAGTTC	NM_204359.2
	R	GAGCTGGGATGGTTTCTGAGTA	
DF	F	TCATTGTGCTAGGTGCCA	L08165
	R	CCTCTTCCAGCCATCTTT	
GH4	F	TGTCACCAACTGGGACGATA	NM_001135968.1
	R	CTGGGTCATCTTTTCACGGT	
293FT	F	GGCCAACCGCGAGAAGA	U20114
	R	CCTCGTAGATGGGCACAGTGT	
Fir	F	TTGGAATCCATCTTGCTCCAA	U47295
	R	TCCGTGCTCCAAAACAACAA	
Ren	F	AGGTGGTAAACCTGACGTTGTACA	AF025845
	F	ATCCTGGGTCCGATTCAATAAAC	

Table S6. Probe sequences for EMSA.

Name	Title	Sequence
WT probe	F	CCTTCAGGCTTGACAGTGACCTCCAGAT
	R	ATCTGGAGGTCACTGTCAAGCCTGAAGG
Mutated probe	F	CCTTCAGGCTTAACAGCACCATCCAGAT
	R	ATCTGGATGGTGCTGTTAAGCCTGAAGG

Sequence with underline was protective bases and enzyme loci.

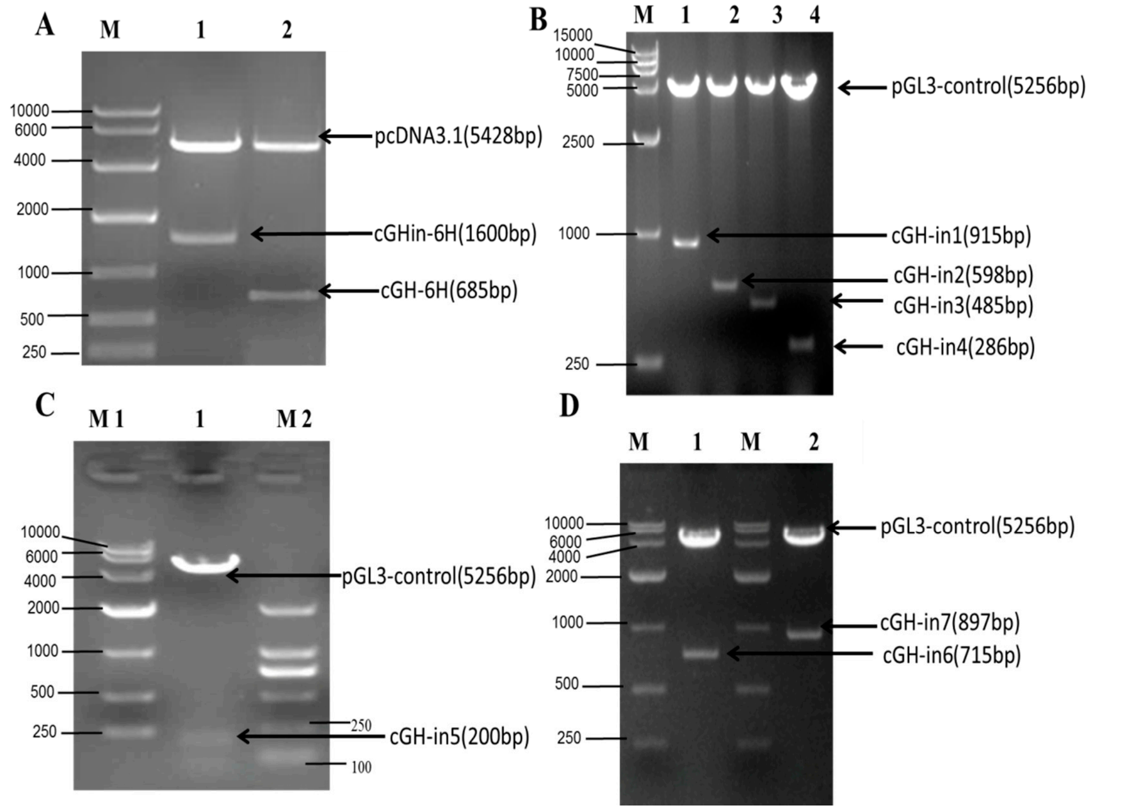

Figure S1. Plasmid identified. (**A**) Gel electrophoresis detection of the pcDNA3.1cGH-6H, pcDNA3.1cGH-in-6H after digestion by *Hin*d III and *Eco*R I. M was Marker; 1, 2 represent degenerated plasmid of pcDNA3.1cGH-6H, pcDNA3.1cGH-in-6H after double enzyme digestion; (**B**) Gel electrophoresis detection of the four plasmids after digestion by *Kpn* I and *Xho* I, M was Marker; 1, 2, 3, 4 represent degenerated plasmid of pGL3-cGH-in1, pGL3-cGH-in2, pGL3-cGH-in3 and pGL3-cGH-in4 after double enzyme digestion, respectively; (**C**) Gel electrophoresis detection of the plasmid pGL3-cGH-in5 after digestion by *Kpn* I and *Nhe* I, M was Marker; 1 represent degenerated plasmid of pGL3-cGH-in5 after double enzyme digestion; (**D**) Gel electrophoresis detection of the two plasmids after digestion by *Kpn* I and *Xho* I, M was Marker; 1, 2 represent degenerated plasmid of pGL3-cGH-in6 and pGL3-cGH-in7 after double enzyme digestion, respectively.

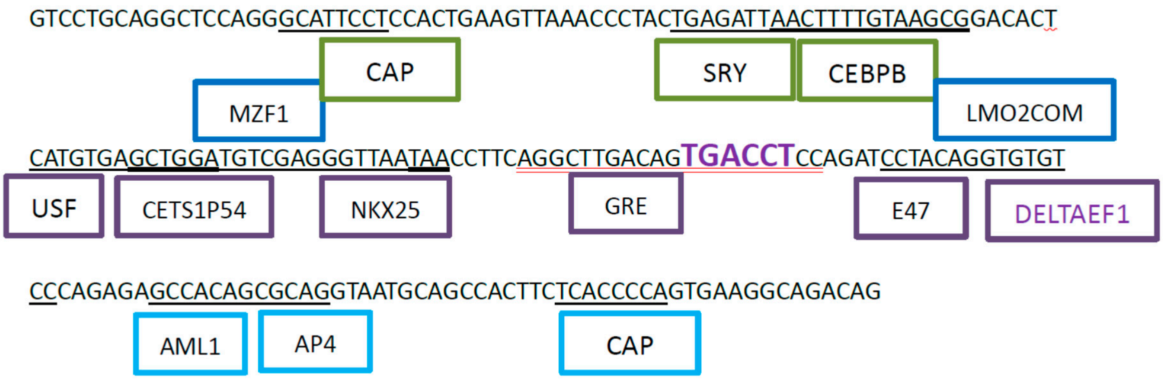

Figure S2. TFBSs in the +485/+684 region of the chicken GH gene. The outlined sequences correspond to the TFBS. Characters in each textbox correspond to the name of each potential element. The putative GRE is double-underlined and the TGACCT motif is indicated by a bold character.
